# Heart Disease: A Word of Comfort

**Published:** 1887-02-26

**Authors:** 


### Heart Disease: A Word of Comfort.

By a Consultant.

Sir Andrew Clark has lately published some lectures in the *British Medical Journal* on the very interesting and, to some, alarming subject of heart-disease. It is not too much to say that to a large number of persons life is little better than a living death because of this terrible bugbear. They who suffer from it know of it; their families and family connections know of it too; and the knowledge is a nightmare, a skeleton in the closet, which banishes every joy, and makes happiness impossible. To many such Sir Andrew Clark has a word, not merely of comfort, but of reasonable confidence and lively hope. There is a prevalent impression of very long standing, that any person who has once become the subject of valvular disease of the heart is thenceforth and for ever in a condition of imminent danger. It is supposed that he is liable to fall down dead at any moment on the slightest provocation. This impression has been strengthened and intensified by the general opinion of medical men and their management of these unhappy cases. In many an instance the patient has been instructed to give up business partially or entirely; to take as much rest as possible; to avoid excitement, even of the mildest kind, and generally to live the monotonous and aimless life of a chronic invalid. Sir Andrew Clark enters a firm protest against both the prevailing opinions and the treatment. Generalising the results of more than thirty years' experience, he concludes that many cases of undoubted valvular disease neither render the patient unfit for business, nor do they place him in any real danger of sudden death, or tend necessarily to an appreciable shortening of life. Contrary to received opinion, he affirms that in a certain proportion of cases he has known the symptoms entirely disappear, and the patient become to all intents and purposes a sound man. He even goes so far as to say that insurance offices may in certain selected cases grant life policies at ordinary rates.

This is a most important contribution to practical medicine, and nothing short of an evangel to unhappy valetudinarians. Of course there are those who will doubt the correctness of these generalisations. To such, it may be answered, that Sir Andrew Clark speaks from a large and varied experience, that he cites numerous cases in proof of his position, and that there has for some time been a growing feeling among careful scientific observers that the prevailing fears and treatment are not justified by facts. The present writer well remembers cases which entirely support the views of Sir Andrew Clark, two of which are here given. Seven or eight years ago Mrs. B——, an old lady of seventy-five, was suffering from slight bronchitis. During the stethoscopic examination of the chest a well-marked murmur was heard at the apex of the heart. "Did you ever suffer from rheumatism?" Mrs. B—— was asked. "I had rheumatic fever when I was twenty-one," was the answer. "Did any medical man ever ask you the question before?" was queried. "Yes, Dr. ——," naming a well-known authority on the heart. A further question was put. "Have you ever suffered from shortness of breath or palpitation?" "Not particularly that I have noticed." This old lady, then seventy-five, was in excellent health, except the severe cold, had never known any special inconvenience from her undoubted valvular disease of fifty-four years' duration, and is probably still living at the age of eighty-one. When she was twenty-two every insurance office in the kingdom would have declined her; and every one of them would have lost an excellent life.

Five years ago, P. L——, a boy of eleven, was suffering from a cold. Precisely as in the case of Mrs. B——, the use of the stethoscope for another purpose accidentally discovered a loud cardiac murmur. The parents were informed of the fact; but were advised to let the boy go to school as usual, only that he was to have a moderate course of study, and as much fresh air as possible, with two or three visits to the sea-side annually. The parents, however, were seriously alarmed, and decided to consult Sir ——, a well-known physician. Sir —— advised that the boy should be taken away from school, kept at rest as much as possible, have an attendant to walk out gently with him once or twice a day, and generally be regarded as a confirmed invalid. The result was soon apparent in a decided flagging of the general health, and a marked exaggeration of all the cardiac symptoms. The writer was thereupon again consulted. He repeated his former advice. The attendant was dismissed, school was resumed; a fair degree of health was restored, and the patient is now a student of King's College, having required neither attendance nor medicine from that day to this.

There is little doubt that in many cases of valvular disease, whether resulting from acute rheumatism or otherwise, the man who knows nothing of his condition will generally be both healthier and happier than he who, being too conscious of his troubles, runs about from one physician to another in chronic and all but groundless terror; whilst it is certain that for most patients thus afflicted, moderate work, plenty of fairly energetic exercise, with simple diet, are more conducive to health and longevity than the old-fashioned treatment of enforced idleness, drugging, and the ceaseless avoidance of dangers which are to a large extent purely imaginary.

